# The Effect of Tacrine on Functional Response of the Lower Oesophageal Sphincter Assessed by Endoscopic Luminal Impedance Planimetry in Experimental Pigs

**DOI:** 10.3390/ph17121588

**Published:** 2024-11-25

**Authors:** Jan Bures, Martin Novak, Vera Radochova, Darina Kohoutova, Lukas Prchal, Jan Martinek, Jan Mares, Jaroslav Cerny, Stepan Suchanek, Jaroslav Pejchal, Barbora Voxova, Petr Urbanek, Miroslav Zavoral, Ondrej Soukup

**Affiliations:** 1 Biomedical Research Centre, University Hospital Hradec Kralove, 500 05 Hradec Kralove, Czech Republic; martin.novak@fnhk.cz (M.N.); darina.kohoutova@rmh.nhs.uk (D.K.); lukas.prchal@fnhk.cz (L.P.); jaroslav.pejchal@unob.cz (J.P.); barbora.voxova@fnhk.cz (B.V.); ondrej.soukup@fnhk.cz (O.S.); 2 Department of Medicine, First Faculty of Medicine, Charles University, Prague and Military University Hospital Prague, 169 02 Prague, Czech Republic; stepan.suchanek@uvn.cz (S.S.); petr.urbanek@uvn.cz (P.U.); miroslav.zavoral@uvn.cz (M.Z.); 3 Institute of Gastrointestinal Oncology, Military University Hospital Prague, 169 02 Prague, Czech Republic; 4 Animal Laboratory, Military Faculty of Medicine, University of Defence, 500 02 Hradec Kralove, Czech Republic; vera.radochova@unob.cz; 5 The Royal Marsden NHS Foundation Trust, London SW3 6JJ, UK; 6 Department of Gastroenterology, St. Anne’s University Hospital Brno, 602 00 Brno, Czech Republic; jan.martinek@volny.cz; 7 Department of Data Science, Institute for Clinical and Experimental Medicine, 140 21 Prague, Czech Republic; janmares42@gmail.com; 8 Section of Medical Information, ANOVA CRO, 160 00 Prague, Czech Republic; jaroslavcerny98@gmail.com; 9 Department of Toxicology and Military Pharmacy, Military Faculty of Medicine, University of Defence, 500 02 Hradec Kralove, Czech Republic; 10 Department of Biological and Medical Sciences, Faculty of Pharmacy, Charles University, 500 03 Hradec Kralove, Czech Republic

**Keywords:** Alzheimer’s disease, endoscopic luminal impedance planimetry, experimental pigs, lower oesophageal sphincter, tacrine

## Abstract

**Background/Objectives:** Tacrine is a centrally active non-competitive reversible acetylcholinesterase inhibitor. It also exerts antagonising activity against *N*-methyl-D-aspartate receptors. Tacrine was approved for the treatment of Alzheimer’s disease in 1993, but was withdrawn from clinical use in 2013 because of its hepatotoxicity and gastrointestinal side effects. Nevertheless, tacrine is currently facing a renewed wave of interest primarily due to several new tacrine-incorporated hybrids and derivates. There were two specific aims for this study: firstly, to explain the mechanisms of the adverse action of tacrine, as a distinctive example of a highly effective acetylcholinesterase inhibitor; and secondly to check whether luminal impedance planimetry is feasible for preclinical testing of possible side effects of compounds potentially toxic to the gastrointestinal tract. **Methods:** Six experimental pigs were used as the animal model in this study. Five major parameters were evaluated: luminal pressure (mmHg), estimated diameter (mm), cross-sectional area (mm^2^), distensibility (mm^2^/mmHg), and zone compliance (mm^3^/mmHg). All measurements were performed before and 360 min after intragastric administration of 200 mg tacrine (at the porcine tacrine T_max_). **Results:** This study consistently demonstrated an increase in luminal pressure (a directly measured indicator) for the particular balloon filling volumes used, and inversely a reciprocal decrease in the other parameters after tacrine administration. **Conclusions:** Endoscopic luminal impedance planimetry is a feasible method to evaluate functional response of the lower oesophageal sphincter to tacrine in experimental pigs. Tacrine did not compromise the function of the lower oesophageal sphincter either toward oesophageal spasms or, in contrast, decreased competence of the lower oesophageal sphincter.

## 1. Introduction

Tacrine (9-amino-1,2,3,4-tetrahydroacridine monohydrochloride) is primarily a centrally acting non-competitive reversible acetylcholinesterase inhibitor [1]. Recently, also its antagonising activity against *N*-methyl-d-aspartate receptors (NMDAR) was proved [2]. However, its mode of action is even more complex, involving cholinergic, GABAergic, nitrinergic, and glutamatergic systems [3]. Tacrine was approved for the treatment of Alzheimer’s disease in 1993 [4]. However, its therapeutic potential was compromised by serious side effects, especially hepatotoxicity, revealed since the early 1990s [5,6,7,8], and peripheral cholinergic effects [9]. For this reason, the drug was withdrawn from clinical use in 2013 [10,11,12]. Apart from serious hepatotoxicity, tacrine has further dose-limiting side effects, including diarrhoea, nausea and vomiting [10]. Recently, tacrine, as a compound effectively acting in several pathways, has received renewed interest due to several new tacrine-incorporated hybrids and derivates [13,14,15,16,17,18]. These compounds retain the positive effect of tacrine while seeking to reduce or even suppress hepatotoxicity [9,10,11,12,13,14,15,16,17,18,19,20,21,22].

The most important factors responsible for proper function of the lower oesophageal sphincter are the tone of the smooth muscles, excitatory cholinergic and inhibitory nitrinergic neurohormonal control [23,24]. Major pathological conditions associated with dysfunction of the lower oesophageal sphincter in humans are gastro-oesophageal reflux disease, achalasia and hypertensive lower oesophageal sphincter [23,24].

Considering the gastrointestinal tract, only sparse experimental data have been published so far, evaluating the effect of tacrine on motor function of the cat oesophagus and on the rat gastric smooth muscle [25,26]. The functional lumen impedance planimetry (FLIP) enables direct measurement of luminal pressure, allowing the assessment of cross-sectional areas, intraluminal diameters, and wall biomechanical properties [27,28,29,30]. Thus, the aim of this experimental study was to evaluate lower oesophageal sphincter characteristics in response to tacrine administration by endoscopic luminal impedance planimetry. There were two specific aims for this study. The first was to elucidate mechanisms of the adverse action of tacrine, as a distinctive example of a highly effective cholinesterase inhibitor, and secondly to check whether luminal impedance planimetry is a feasible method for preclinical testing of possible side effects of potentially (sub)toxic compounds.

## 2. Results

**Figure 1:**
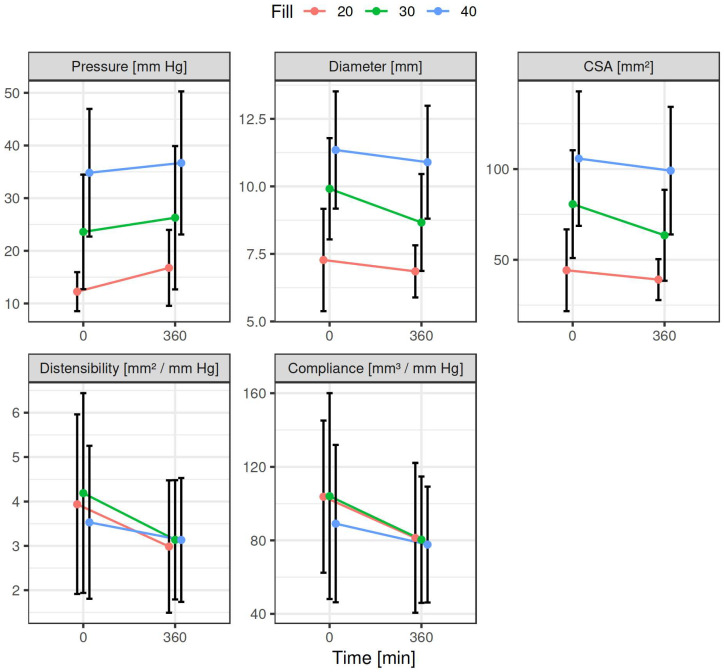
Endoscopic luminal impedance planimetry of the porcine lower oesophageal sphincter. The line plot shows the mean values of parameters (across the six animals) at times 0 and 360 min, along with error bars given by ±one standard deviation for the three different filling volumes. Time 0: time before and Time 360: 360 min after intragastric administration of 200 mg tacrine; CSA: cross-sectional area; Filling: balloon filling volumes 20 mL, 30 mL and 40 mL.

**Figure 2:**
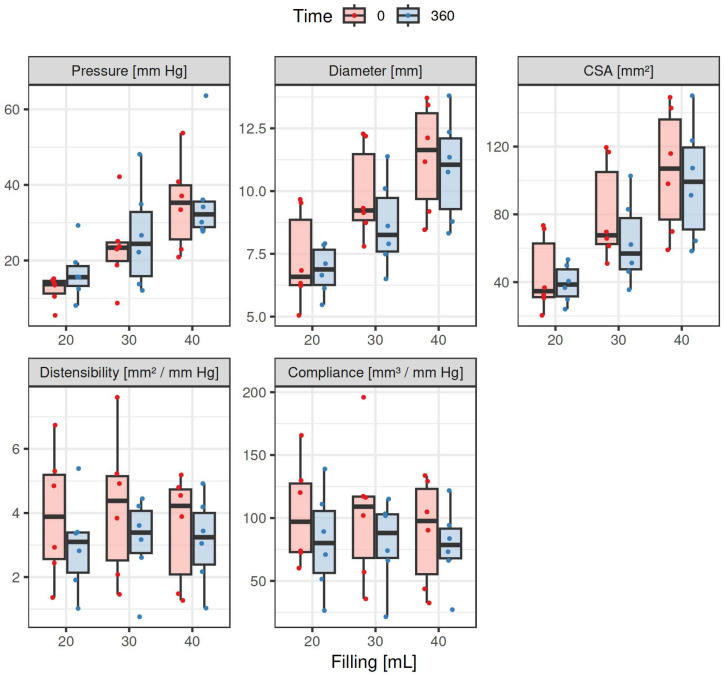
Endoscopic luminal impedance planimetry of the porcine lower oesophageal sphincter. Box-plots show the medians (thick horizontal lines), the first and the third quartiles (span of the boxes), and the range of the data without outliers (vertical lines). CSA: cross-sectional area; Fill: balloon filling volumes 20 mL, 30 mL and 40 mL.

All measurements of luminal impedance planimetry were successfully performed. Results of all the parameters from particular experimental pigs are listed in Table 1. A summary of descriptive statistics of particular parameters of six animals is given in Table 2. Results of the statistical test of changes in measured values before and after intragastric administration of 200 mg tacrine are presented in Table 3. Data showing differences between Time 0 (basal values) and Time 360 min (values at porcine tacrine T_max_), for all three balloon filling volumes (20, 30 and 40 mL) are summarised in Figure 1 and Figure 2. The line plot shows the mean values of parameters (across the six subjects) at times 0 and 360 min along with error bars given by ±one standard deviation for the three different filling volumes. The points are connected for visual aid (Figure 1). Presented are box-plots showing the medians (thick horizontal lines), the first and the third quartiles (span of the boxes), and the range of the data without outliers (vertical lines). Also, all the data points are plotted (Figure 2). The results consistently demonstrated an increase in luminal pressure for the particular filling volumes used, and inversely a reciprocal decrease in the other parameters after intragastric tacrine administration.

**Figure 3:**
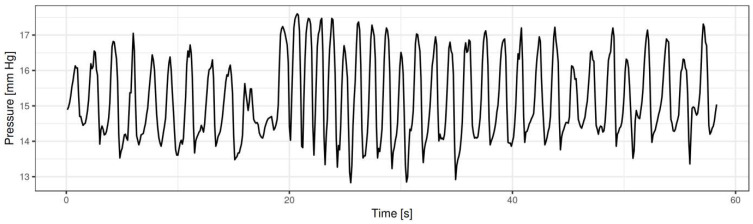
Endoscopic luminal impedance planimetry of the porcine lower oesophageal sphincter. Example of a single measurement of pressure of one experimental pig. Several readings were necessary to obtain a stabilised average value.

The sample rate of FLIP is 10 Hz, i.e., ten readings per second. Different numbers of readings for particular parameters were necessary to obtain a stabilised average. These figures are displayed in Table 1. The apparatus can separately provide values taken from particular sensors (in log files). Balloon pressure was measured directly, and the remaining parameters were derived by calculation. Balloon filling volumes and time intervals were recorded as well. The resulting stabilised average is a single figure; no standard deviation is provided by the dedicated software. A single measurement of balloon pressure of one experimental pig is displayed as an example in Figure 3.

## 3. Discussion

This experimental study proved that luminal impedance planimetry is a feasible method to assess the functional response of the lower oesophageal sphincter to administration of tacrine. To the best of our knowledge, this is the first trial studying the impact of any cholinesterase inhibitor on the lower oesophageal sphincter using FLIP in an experimental setting. Luminal pressure (a directly measured indicator) increased while the other parameters decreased upon tacrine administration. All these markers are comparable with normal values in humans [27,28,29,30]. The most important parameters are distensibility and cross-sectional area. These decreased after tacrine administration, but nevertheless they remained within a normal range. Indeed, it seems that tacrine did strengthen the lower oesophageal sphincter but did not stimulate a trend of spasms of the oesophagus. Values after tacrine administration with a 20 mL distension volume are borderline in our study. In humans, a normal value of distensibility is about five to six. A cut-off of 2 mm^2^/mm Hg is recommended and lower figures can be considered as abnormal [29]. Values of cross-sectional area in humans are dependent on balloon filling volumes [27]. We did not use 50 mL or 60 mL distension volumes because of the smaller size of the porcine oesophagus (compared to that of humans). Cross-sectional areas did not increase after tacrine administration in our study. This clearly means that tacrine did not compromise competence of the lower oesophageal sphincter, i.e., it did not increase a risk of gastro-oesophageal reflux disease. The maximum porcine diameter of the lower oesophageal sphincter is lower than the human diameter [27], which can be explained by the lower size of the porcine oesophagus. An increase in pressure after tacrine administration is an anticipated result.

With a standard sample size for experimental animal studies of six subjects, there is only a very limited statistical power to demonstrate statistically significant differences while correcting for multiplicity of testing. The effect sizes would have to be extreme, beyond what can be expected in our settings.

Since endoscopic luminal impedance planimetry is a novel method in the experimental setting, only a few relevant papers have so far been published [31,32,33,34,35,36,37]. Consequently, we cannot at this time compare our current results with other studies.

The experimental pig is a suitable model for preclinical studies of the impact of different drugs on gastrointestinal motility [38,39,40,41]. In our previous projects, we studied myoelectric activity of the porcine stomach after exposure to cholinergics and anticholinergics [4,42,43], and drugs used for the treatment of Alzheimer’s disease, i.e., galantamine, donepezil, rivastigmine and memantine [44,45,46,47]. We used electrogastrography (EGG) to compare the effect of tacrine and 7-methoxytacrine (7-MEOTA) in an experimental porcine model [22]. Tacrine and 7-MEOTA have different impacts on EGG. Tacrine decreased the dominant frequency and induced long-lasting gastric arrhythmia, whereas 7-MEOTA caused a short-term late increase in EGG power in pigs [22].

In the current study, we used a single dose of 200 mg tacrine. This dose was chosen as 2.5 times higher compared to a standard dosage in humans (80 mg/day) [48], mainly to reflect the slower gastric emptying [49,50], and by that ensure a sufficient dose. Such a dose is still non-toxic, but enables highlighting of possible dose-dependent adverse effects.

Endoscopy of experimental pigs is more demanding compared to that in humans. The distance between incisors and the cardia is one-third longer, each animal has a pharyngeal diverticulum (a feature of normal anatomy), and there is a short length of the lesser curvature between the porcine gastric cardia and pylorus with an acute angle. Nevertheless, endoscopy, introduction of catheters and luminal planimetry were feasible and uneventful in all animals. An endotracheal cannula, securing spontaneous breathing, did not compromise either endoscopy or measurements. There was a substantial inter-individual variability in particular animals. Luminal impedance planimetry represents a highly dynamic examination (as demonstrated by short video recordings in Supplementary Materials). The sample rate is 10 Hz, so that ten measurements are accomplished in one second. Several hundred readings (usually for 40–120 s) were necessary for each balloon filling volume in order to obtain a stabilised average for particular parameters. A detailed, exact evaluation of particular parameters was possible owing to the dedicated software. 

The major strength of our current project lies in the first systematic measurements of all major FLIP parameters after administration of an acetylcholinesterase inhibitor in the experimental setting. Secondly, particular basal values before tacrine administration are comparable with those in adult humans [27,28,29,30].

One of the limitations of the current study is that it was accomplished on pigs without any previous intervention, either experimental models of achalasia or gastro-oesophageal reflux disease. That is why our translation assumption toward these diseases has only indirect support. Furthermore, only young adult animals were used, and it is possible that particular parameters could change during aging. Similarly, there may be a sex-related aspect which could not be observed, as all endoscopic luminal impedance planimetry was carried out on female pigs. According to our previous experimental study of porcine oesophageal manometry [51], peristaltic wave pressure was significantly higher in female animals compared to male pigs, and there was also a clearly distinct trend in other motility parameters in favour of the female [51]. A similar sex-related difference in oesophageal motility has been reported in healthy humans, too [52,53]. Although sex-related differences in oesophageal motility are considered to have no clinical significance in humans, they might be taken into consideration when interpreting oesophageal motility tests [52].

## 4. Materials and Methods

### 4.1. Animals

Six experimental female adult pigs (3-month-old) were used in this study (*Sus scrofa* f. *domestica*, hybrids of Czech White and Landrace breeds; mean weight 32.9 ± 3.5 kg). The animals were purchased from a certified breeder (Stepanek, Dolni Redice, Czech Republic; SHR MUHO 2050/2008/41). The pigs were stabled in an accredited animal laboratory (University of Defence, Military Faculty of Medicine, Hradec Kralove). During a two-week acclimatisation, all animals were fed with standard assorted A1 food (Ryhos, Novy Rychnov, Czech Republic) in equal amounts twice a day, and had free access to drinking water. All experiments were performed in the morning on overnight fasting animals under general anaesthesia. Drugs used for induction of anaesthesia were medetomidine 0.1 mg/kg i.m. (Orion Corporation, Espoo, Finland), butorphanol 0.3 mg/kg i.m. (Richter Pharma AG, Wels, Austria) and midazolam 0.3 mg/kg i.m. (Accord Healthcare, London, UK). Subsequent general anaesthesia was maintained by i.v. propofol (Fresenius Kabi Canada, Toronto, ON, Canada), with repeated 1 mL boluses of 20 mg. The pigs were intubated and breathing spontaneously. Vital signs were secured by pulse oximetry.

### 4.2. Design of the Study

**Figure 4:**
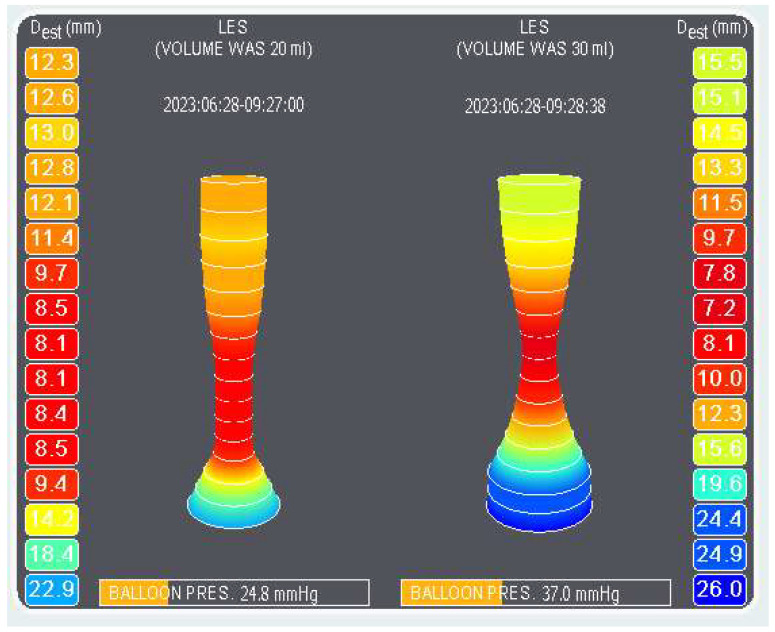
Impedance planimetry of the lower oesophageal sphincter. Filling volumes 20 mL (on the **left**) and 30 mL (on the **right**). Balloon pressure (mm Hg).

Endoscopic luminal impedance planimetry was accomplished using the EndoFLIP 1.0 System (Medtronic, Minneapolis, MN, USA). Impedance catheters were calibrated (pressure-zeroed to atmospheric pressure). Catheters (EF-325N; length of measuring zone 80 mm; 16 paired impedance planimetry sensors) were introduced into the porcine oesophagus and stomach endoscopically by means of a 20 mm snare (diameter 2.3 mm; Micro-Tech Endoscopy, Ann Arbor, MI, USA). A video-gastroscope GIF-Q180 (Olympus Optical Co., Tokyo, Japan) was used, dedicated to animal experiments only. Balloon filling volumes of 20, 30 and 40 mL were employed. The still image in Figure 4 and short real-time video recordings demonstrate impedance planimetry investigations (Supplementary Materials). Five major parameters were evaluated for each filling volume: pressure (mm Hg), estimated diameter (mm), cross-sectional area (mm^2^), distensibility defined as cross-sectional area divided by balloon distending pressure (mm^2^/mm Hg), and zone compliance, defined as the change in volume over a 2 cm long segment spanning five electrodes, centred around the gastro-oesophageal junction (mm^3^/mm Hg). All measurements were performed before and 360 min after administration of 200 mg tacrine (tetrahydroaminoacridine hydrochloride hydrate; purchased from Sigma-Aldrich, Merck Group, Burlington, MA, USA). Tacrine was delivered into the stomach endoscopically. The interval of 360 min was decided based on the porcine tacrine T_max_ (Novak et al., 2024 [54]). The sample rate was 10 Hz. All parameters were read by dedicated software. 

### 4.3. Statistical Analysis

The data are described using medians with quartiles or means with standard errors of the mean. Changes in parameters were tested using a mixed-effect linear model: for each variable, all data points (18 per animal for the three filling volumes) were combined into a single linear mixed model for differences with random effects of subject and filling volume. The intercept coefficients (estimates of the differences) are presented. A positive coefficient indicates an increase, and a negative coefficient a decrease. The resulting *p*-values were further corrected for multiple testing using the Holm method.

### 4.4. Ethics

The project was approved by the Institutional Review Board of the Animal Care Committee (Protocol Number MO 652499/2023-2994). The study was conducted in accordance with the policy for experimental studies [55]. Animals were held and treated in conformity with the European Convention for the Protection of Vertebrate Animals [56].

## 5. Conclusions

Endoscopic luminal impedance planimetry is a feasible method to evaluate functional response of the lower oesophageal sphincter to tacrine in experimental pigs. Our study demonstrated an increase in luminal pressure and, inversely, a reciprocal decrease in the remaining parameters after intragastric tacrine administration. The most important parameters (distensibility and cross-sectional area) remained within a normal range. Tacrine did not compromise the function of the lower oesophageal sphincter either toward oesophageal spasms or, by contrast, decreased competence of the lower oesophageal sphincter.

## Figures and Tables

**Table 1:** Endoscopic luminal impedance planimetry of the lower oesophageal sphincter in particular experimental pigs.

TIME 0 min. **ANIMAL 1**						
Filling	Pressure	Diameter	CSA	Distensibility	Compliance	No meas
20 mL	15.19	9.67	73.42	4.85	120.29	582
30 mL	23.75	12.19	116.76	4.92	116.40	470
40 mL	37.09	13.43	142.69	3.89	90.34	833
TIME 360 min.						
Filling	Pressure	Diameter	CSA	Distensibility	Compliance	No meas
20 mL	15.59	7.11	40.58	2.82	70.94	1172
30 mL	34.90	11.38	102.65	3.17	74.06	350
40 mL	36.06	13.80	150.00	4.19	94.22	215
TIME 0 min. **ANIMAL 2**						
Filling	Pressure	Diameter	CSA	Distensibility	Compliance	No meas
20 mL	10.50	6.23	30.66	2.93	72.57	2433
30 mL	18.79	9.32	69.61	3.84	102.02	1150
40 mL	22.97	12.12	115.88	5.19	129.30	858
TIME 360 min.						
Filling	Pressure	Diameter	CSA	Distensibility	Compliance	No meas
20 mL	12.48	7.91	53.30	5.39	139.08	439
30 mL	13.74	7.49	46.30	3.61	101.88	780
40 mL	28.39	8.32	58.34	2.17	66.22	475
TIME 0 min. **ANIMAL 3**						
Filling	Pressure	Diameter	CSA	Distensibility	Compliance	No meas
20 mL	5.48	6.84	36.76	6.74	165.67	999
30 mL	8.73	9.14	65.79	7.61	195.95	875
40 mL	20.90	11.17	98.04	4.80	133.84	1605
TIME 360 min.						
Filling	Pressure	Diameter	CSA	Distensibility	Compliance	No meas
20 mL	8.10	5.47	24.07	3.37	111.07	1116
30 mL	12.08	7.90	51.33	4.45	115.16	832
40 mL	30.15	10.76	91.28	3.05	73.20	837
TIME 0 min. **ANIMAL 4**						
Filling	Pressure	Diameter	CSA	Distensibility	Compliance	No meas
20 mL	14.01	6.33	32.55	2.44	73.96	1005
30 mL	25.11	7.80	50.99	2.08	56.96	593
40 mL	40.84	8.46	59.05	1.48	43.65	760
TIME 360 min.						
Filling	Pressure	Diameter	CSA	Distensibility	Compliance	No meas
20 mL	19.48	6.65	36.75	1.91	51.44	599
30 mL	26.67	8.61	62.20	2.61	66.32	682
40 mL	34.18	11.35	107.30	3.44	83.68	2064
TIME 0 min. **ANIMAL 5**						
Filling	Pressure	Diameter	CSA	Distensibility	Compliance	No meas
20 mL	14.80	5.05	20.40	1.36	60.14	915
30 mL	42.17	8.74	61.35	1.46	35.71	1056
40 mL	53.72	9.19	69.89	1.27	32.45	492
TIME 360 min.						
Filling	Pressure	Diameter	CSA	Distensibility	Compliance	No meas
20 mL	29.28	6.13	29.85	1.02	26.41	1380
30 mL	48.10	6.50	35.50	0.76	21.46	588
40 mL	63.61	8.79	64.35	1.03	27.12	871
TIME 0 min. **ANIMAL 6**						
Filling	Pressure	Diameter	CSA	Distensibility	Compliance	No meas
20 mL	13.48	9.53	71.51	5.31	129.91	663
30 mL	23.02	12.28	119.52	5.23	117.32	700
40 mL	33.44	13.71	149.02	4.55	104.95	1176
TIME 360 min.						
Filling	Pressure	Diameter	CSA	Distensibility	Compliance	No meas
20 mL	15.64	7.85	49.86	3.40	89.27	1178
30 mL	22.21	10.10	83.00	4.22	103.37	858
40 mL	27.74	12.35	123.51	4.92	121.87	1612

TIME 0 min.: before intragastric administration of 200 mg tacrine; TIME 360 min.: measurement 360 min. after tacrine administration. Filling: balloon filling volume (mL); Pressure: balloon pressure (mm Hg); Diameter: estimated diameter (mm); CSA: cross-sectional area (mm^2^); Distensibility (mm^2^/mm Hg); Compliance: zone compliance (mm^3^/mm Hg); No Meas: number of measurements necessary to obtain stabilised average value.

**Table 2:** Summary of descriptive statistics of particular parameters of endoscopic luminal impedance planimetry of the lower oesophageal sphincter in six experimental pigs.

Variable	Mean	SD	SEM	Median	Q1	Q3
Pressure [mm Hg] 20 mL 0 min.	12.24	3.71	1.51	13.75	11.25	14.60
Pressure [mm Hg] 20 mL 360 min.	16.76	7.21	2.94	15.62	13.26	18.52
Pressure [mm Hg] 30 mL 0 min.	23.59	10.88	4.44	23.38	19.85	24.77
Pressure [mm Hg] 30 mL 360 min.	26.28	13.61	5.56	24.44	15.86	32.84
Pressure [mm Hg] 40 mL 0 min.	34.83	12.12	4.95	35.26	25.59	39.90
Pressure [mm Hg] 40 mL 360 min.	36.69	13.59	5.55	32.16	28.83	35.59
Diameter [mm] 20 mL 0 min.	7.28	1.89	0.77	6.58	6.26	8.86
Diameter [mm] 20 mL 360 min.	6.85	0.96	0.39	6.88	6.26	7.66
Diameter [mm] 30 mL 0 min.	9.91	1.87	0.77	9.23	8.84	11.47
Diameter [mm] 30 mL 360 min.	8.66	1.79	0.73	8.25	7.59	9.73
Diameter [mm] 40 mL 0 min.	11.35	2.17	0.89	11.64	9.69	13.10
Diameter [mm] 40 mL 360 min.	10.89	2.09	0.85	11.05	9.28	12.10
CSA [mm^2^] 20 mL 0 min.	44.22	22.54	9.20	34.66	31.13	62.82
CSA [mm^2^] 20 mL 360 min.	39.07	11.28	4.61	38.66	31.58	47.54
CSA [mm^2^] 30 mL 0 min.	80.67	29.70	12.12	67.70	62.46	104.97
CSA [mm^2^] 30 mL 360 min.	63.50	25.07	10.23	56.76	47.56	77.80
CSA [mm^2^] 40 mL 0 min.	105.76	37.06	15.13	106.96	76.93	135.99
CSA [mm^2^] 40 mL 360 min.	99.13	35.17	14.36	99.29	71.08	119.46
Distensibility [mm^2^/mm Hg] 20 mL 0 min.	3.94	2.02	0.83	3.89	2.56	5.20
Distensibility [mm^2^/mm Hg] 20 mL 360 min.	2.98	1.49	0.61	3.09	2.14	3.39
Distensibility [mm^2^/mm Hg] 30 mL 0 min.	4.19	2.25	0.92	4.38	2.52	5.15
Distensibility [mm^2^/mm Hg] 30 mL 360 min.	3.14	1.35	0.55	3.39	2.75	4.07
Distensibility [mm^2^/mm Hg] 40 mL 0 min.	3.53	1.72	0.70	4.22	2.08	4.74
Distensibility [mm^2^/mm Hg] 40 mL 360 min.	3.13	1.40	0.57	3.24	2.39	4.00
Compliance [mm^3^/mm Hg] 20 mL 0 min.	103.76	41.36	16.89	97.12	72.92	127.51
Compliance [mm^3^/mm Hg] 20 mL 360 min.	81.37	40.74	16.63	80.10	56.31	105.62
Compliance [mm^3^/mm Hg] 30 mL 0 min.	104.06	55.98	22.85	109.21	68.22	117.09
Compliance [mm^3^/mm Hg] 30 mL 360 min.	80.38	34.38	14.04	87.97	68.25	103.00
Compliance [mm^3^/mm Hg] 40 mL 0 min.	89.09	42.77	17.46	97.65	55.32	123.21
Compliance [mm^3^/mm Hg] 40 mL 360 min.	77.72	31.53	12.87	78.44	67.97	91.58

SD: standard deviation; SEM: standard error of the mean; Q1: first quartile; Q3: third quartile; 0 min.: time before; 360 min.: time after intragastric administration of 200 mg tacrine; balloon filling volumes 20 mL, 30 mL and 40 mL; CSA: cross-sectional area.

**Table 3:** Results of the statistical test of changes in measured values before and after intragastric administration of 200 mg tacrine.

Variable	Coefficient	Standard Error	*p*-Value (Corrected)
Pressure [mm Hg]	3.02	1.71	0.678
Diameter [mm]	−0.71	0.44	0.678
CSA [mm^2^]	−9.65	6.84	0.678
Distensibility [mm^2^/mm Hg]	−0.80	0.47	0.678
Compliance [mm^3^/mm Hg]	−19.15	10.77	0.678

The intercept coefficients (estimates of the differences) are presented. Positive coefficient indicates an increase, negative coefficient a decrease. The resulting *p*-values were further corrected for multiple testing using the Holm method. CSA: cross-sectional area.

## Data Availability

All data generated or analysed during this study are included in this article.

## Supplementary Materials

The following supporting information can be downloaded at: https://www.mdpi.com/article/10.3390/ph17121588/s1. Video S1: Impedance planimetry is a highly dynamic examination. Several hundred readings (usually lasting 40–120 s) are necessary for each balloon filling volume to obtain a stabilised average value. Expansion volume of 30 mL was used at this particular measurement. Video S2: Short video sequence as an example of impedance planimetry of the porcine lower oesophageal sphincter on a 40 mL balloon filling volume.
