# Supplementary material for: The Effect of Tacrine on Functional Response of the Lower Oesophageal Sphincter Assessed by Endoscopic Luminal Impedance Planimetry in Experimental Pigs
Source: Pharmaceuticals (Basel). 2024 Nov 25;17(12):1588. doi: 10.3390/ph17121588 (PMC11678239; doi:10.3390/ph17121588)
Supplement: Supplementary file 1 [file pharmaceuticals-17-01588-s001.zip › pharmaceuticals-3266659_legend.pdf]

Supplementary Material

Video 1:
Impedance planimetry is a highly dynamic examination. Several hundred readings (usually lasting 40 - 120 seconds) are necessary for each balloon filling volume to obtain a stabilized average value. Expansion volume of 30 mL was used at this particular measurement.
Video 2:
Short video sequence as an example of impedance planimetry of the porcine lower oesophageal sphincter on a 40-mL balloon filling volume.
